# The *MYC* Road to Hearing Restoration 

**DOI:** 10.3390/cells1040667

**Published:** 2012-09-25

**Authors:** Benjamin Kopecky, Bernd Fritzsch

**Affiliations:** 1 Department of Biology, 143 Biology Building, University of Iowa, Iowa City, IA 52242, USA; Email: bernd-fritzsch@uiowa.edu; 2 Medical Scientist Training Program, Carver College of Medicine, University of Iowa, Iowa City, IA 52242, USA

**Keywords:** development, cochlea, N-Myc, L-Myc, cell cycle, stem cell

## Abstract

Current treatments for hearing loss, the most common neurosensory disorder, do not restore perfect hearing. Regeneration of lost organ of Corti hair cells through forced cell cycle re-entry of supporting cells or through manipulation of stem cells, both avenues towards a permanent cure, require a more complete understanding of normal inner ear development, specifically the balance of proliferation and differentiation required to form and to maintain hair cells. Direct successful alterations to the cell cycle result in cell death whereas regulation of upstream genes is insufficient to permanently alter cell cycle dynamics. The *Myc* gene family is uniquely situated to synergize upstream pathways into downstream cell cycle control. There are three *Mycs* that are embedded within the *Myc/Max/Mad* network to regulate proliferation. The function of the two ear expressed *Mycs*, *N-Myc* and *L-Myc* were unknown less than two years ago and their therapeutic potentials remain speculative. In this review, we discuss the roles the *Mycs* play in the body and what led us to choose them to be our candidate gene for inner ear therapies. We will summarize the recently published work describing the early and late effects of *N-Myc* and *L-Myc* on hair cell formation and maintenance. Lastly, we detail the translational significance of our findings and what future work must be performed to make the ultimate hearing aid: the regeneration of the organ of Corti.

## 1. Introduction

### Hearing Loss Is a Worldwide Problem with No Perfect Solution

**Figure 1:**
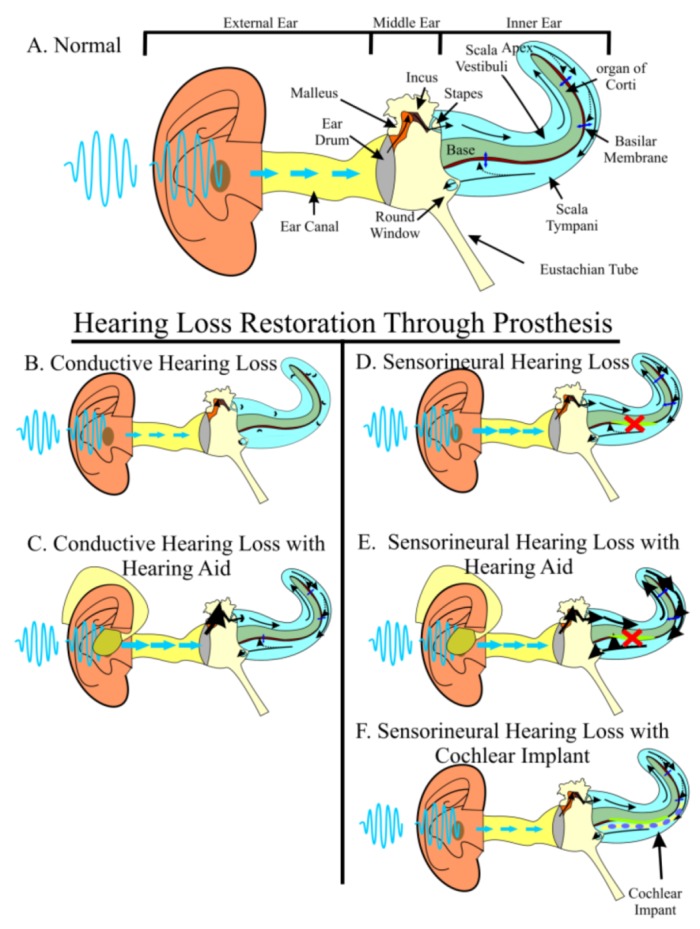
Hearing requires the precise stimulation of hair cells. Sound waves from the environment are funneled into the auditory canal by the external ear. Sound waves hit the ear drum and are turned into physical vibrations that are propagated through three middle ear bones, the malleus, the incus, and the stapes. The stapes articulates with the oval window, which separates the middle ear from the inner ear’s fluid-filled scala vestibuli. Physical vibrations of the middle ear bones are converted into compression and rarefaction of the perilymphatic fluid. The scala vestibuli is continuous with the scala tympani. As the fluid waves propagate through the scalae, they cause vibrations of the overlaying basilar membrane. This vibration results in a shearing force of organ of Corti hair cells against the tectorial membrane to activate the auditory nerve (**A**). Light blue arrows indicate sound wave movement. Black arrows indicate vibration and fluid movement. Dark blue arrows indicate basilar membrane vibration. In conductive hearing loss (**B**), sound waves are not accurately propagated to the inner ear. Hearing aids (**C**) can amplify sound waves to overcome this dampening effect. In sensorineural hearing loss (**D**), hair cells are lost. Hearing aids (**E**) can partially overcome the resulting hearing deficit by increasing the amplitude to nearby cells; however, no hearing aid can stimulate lost hair cells and furthermore, hearing aids may create discomfort by over-stimulating remaining hair cells. The best current treatment for sensorineural hearing loss is cochlear implants (**F**) which directly stimulate auditory neurons through an electrode array surgically implanted into the inner ear.

Hearing loss afflicts over 500 million people worldwide, including half of individuals over the age of 65 and 3 in every 1,000 births totaling 16 million children [1], making hearing loss the most common neurosensory disorder. Aside from these staggering figures, individuals with hearing loss carry heavy social, economic, and quality of life burdens [2]. In normal hearing individuals, sound waves enter the external ear canal to cause physical vibrations of the tympanic membrane (ear drum). These vibrations are propagated through the three middle ear ossicles, the last of which, the stapes, articulates with the perilymphatic fluid-filled scalae of the inner ear. The vibrations of the stapes cause perilymphatic fluid pressure waves to vibrate the basilar membrane. The basilar membrane is the support for the organ of Corti supporting cells and hair cells. Combined, they physically and ionically separate the scala tympani and the endolymphatic fluid-filled scala media. Vibrations of the basilar membrane move organ of Corti hair cells against the tectorial membrane to provide a shearing force. The shearing force results in opening of ion channels at the tips of stereocilia and depolarization of hair cells. Depolarization mediated transmitter release ultimately generates depolarizations on neurosensory afferents that send neural impulses through the spiral ganglion neurons to the cochlear nucleus of the brainstem and eventually to the auditory cortex, allowing those initial sound waves to be interpreted as meaningful stimuli (Figure 1A). Hearing loss occurs when sound waves from the environment are unable to sufficiently stimulate the organ of Corti hair cells to send neural impulses to the brain (conductive hearing loss) or if hair cells are lost (sensorineural hearing loss).

Conductive and sensorineural hearing losses (SNHL) both affect the stimulation of organ of Corti hair cells, but in different ways. In cases of conductive hearing loss, sound waves are hindered from reaching the hair cells, often through increased resistance in the middle ear, as is the case in otosclerosis or otitis media (Figure 1B). Because of the resulting decreased amplitude of sound waves reaching the inner ear, conductive hearing loss can be partially overcome with either hearing aids, an auditory prosthesis in which sound waves are amplified prior to entering the ear canal (Figure 1C), or surgery, to mitigate the impedance. On the other hand, SNHL often results from the loss of organ of Corti hair cells. In SNHL, sound waves enter the ear canal and are transformed into physical vibrations of normal amplitude; however, damaged hair cells are unable to respond to the vibrations and fail to stimulate ganglion neurons (Figure 1D). In contrast to non-mammalian species, human SNHL is irreversible [3,4], meaning that the hair cells we were born with are all the hair cells we will ever have; thus, it is important to understand how hair cells are lost and what can be done to restore functionality.

Organ of Corti hair cell damage can be grouped as either inherited or acquired. Inherited hearing loss occurs when genes necessary for normal hair cell formation are mutated, resulting in either developmental malformations of the organ of Corti hair cells or predisposition to hair cell damage. Mutations can also occur in genes necessary for the development and function of the tectorial membrane, hair cell synapses, spiral ganglion, and ionic composition Early detection (OtoSCOPE; [5]) is now possible for some syndromic and non-syndromic hearing loss mutations [6]; however, there are several hundred unique syndromes with hearing loss as a symptom [7] and none have an available intervention to prevent their onset. Ultimately, inherited hearing loss must be treated as early as possible with personalized treatment to ensure near-normal, lifelong communication and language development [8,9]. 

Individuals without any known genetic defect may also lose hair cells due to an accumulation of acquired environmental insults, ranging from loud noises to ototoxic drugs to aging. Excluding local sound effects, hair cell loss typically progresses from base to apex (high to low frequency) and from outer to inner hair cells. In a restricted number of cases, hair cell loss may be partially prevented with hearing protection such as ear plugs. In the majority of cases, the damage is unexpected, cumulative, or otherwise unpreventable (for example cisplatin treatment). In summary, SNHL is prevalent, irreversible, multi-factorial, and mostly unpreventable given human habits, leading to a global problem affecting hundreds of millions of people without bias towards race, ethnicity, or gender. 

Patients suffering from SNHL have few options. Depending on the degree of hair cell loss, hearing aids may have limited benefit as no increase in amplitude is able to stimulate lost hair cells (Figure 1E). Furthermore, because hearing aids mostly increase indiscriminately the amplitude to the ear, this may create additional damage to the remaining hair cells or otherwise cause patient discomfort. Some modern hearing aids can be digitally programmed to provide somewhat discriminated amplification avoiding to an extent this problem. Other hearing prostheses such as the bone anchored hearing aid (BAHA) [10,11] or direct acoustic cochlear stimulation (DACS) [12] are often insufficient. The best of the current treatments for patients with neurosensory hearing loss are cochlear implants, a prosthesis that is surgically inserted into the scala tympani of the inner ear and stimulates nearby spiral ganglion neuron processes (Figure 1F). While cochlear implants have proven beneficial to patients, they have several limitations. The normal organ of Corti has over 15,000 hair cells that are organized into one row of inner hair cells plus three rows of outer hair cells aligned along the two-and-a-half turn cochlea such that a single set of hair cells responds to a narrow frequency range enabling high frequency sound resolution from 2–20 kHz in humans, and up to 61 kHz in mouse [13]. In patients with cochlear implants, only a small set (as little as 6–8) of electrodes are used to recapitulate the 15,000 hair cells and although individual performance does improve over time, patients suffer under many listener environments, are never able to fully appreciate various tones [14,15,16,17,18], and their hearing age never reaches their chronological age; however, the younger the patient, the better the outcome [19,20]. Furthermore, without proper neurotrophic factors provided by hair cells to the neurons, neurons may be progressively lost, as they are in animal models [21,22], but limited human data indicate otherwise [23]. This makes research into long-term neuronal survival crucial for continued success of cochlear implants [24]. Together, current prostheses are unable to provide a long-term solution capable of perfect hearing restoration, relegating the “world’s best hearing aid” to regeneration of the damaged or lost organ of Corti [25]. 

## 2. The Two-Pronged Attack of Hearing Loss Treatment

### 2.1. Prevention of Hearing Loss as a First-Line Treatment

There are two avenues that may ultimately prove to be of help for hearing loss: first, prevention of hair cell loss, and secondly, regeneration of damaged or lost hair cells. The best treatment for any syndrome is to prevent the onset of symptoms. Hearing loss is no different. Currently, certain acquired hair cell losses are mostly unpreventable (cisplatin treatment, for example); but, prevention may one day be possible through increasing the resilience of hair cells’ natural maintenance mechanisms. *Atoh1* is known to be necessary for hair cell formation [26] and in the absence of *Atoh1* [27], with only transient expression of *Atoh1* [28], or in the absence of *Atoh1* downstream genes *Pou4f3* [2,29,30] and *Barhl1* [31], hair cells are lost. Manipulation of these or other yet-to-be determined genes may prove to be sufficient to prevent organ of Corti hair cell death and subsequent hearing loss. Additionally, other avenues to prevent hair cell death through manipulation of reactive oxygen species, which may play a role in cisplatin treatment for example, are being explored [32]. 

### 2.2. Regeneration of Hair Cells when Prevention Fails

In cases where hair cell loss is not prevented, hair cell regeneration may be the avenue of choice and would likely be achieved through targeted proliferation followed by the directed differentiation of either stem cells or inner ear cells, such as supporting cells [33,34]. In non-mammalian species, damage to hair cells is swiftly responded to through re-initiation of proliferation of supporting cells [35,36,37,38,39]. There are two theories how this re-entry of a supporting cell replaces the lost hair cell. Either, cell cycle re-entry of the supporting cell dedifferentiates the supporting cell to form a neurosensory precursor cell which then undergoes cellular division to form two cells with subsequent differentiation into one hair cell and one supporting cell. Or, the cell cycle re-entry of the supporting cell forms two supporting cells (with no dedifferentiation) with one of the supporting cells transdifferentiating into a hair cell. Transdifferentiation is the process of directly transforming from one differentiated cell type into a unique second differentiated cell type and has been shown to be possible using various molecular means [40,41,42]. Regardless, since mammalian inner ear cells are unable to re-enter the cell cycle, transforming supporting cells into hair cells would lead to a depletion of supporting cells. Since transformed supporting cells cannot sustain newly formed hair cells, proliferation is required for mammalian hair cell regeneration to provide enough cellular material for both supporting cell and hair cell differentiation. Unfortunately, current attempts at forced cell cycle re-entry of differentiated cells results in cells have limited viability [43,44,45,46].

**Figure 2:**
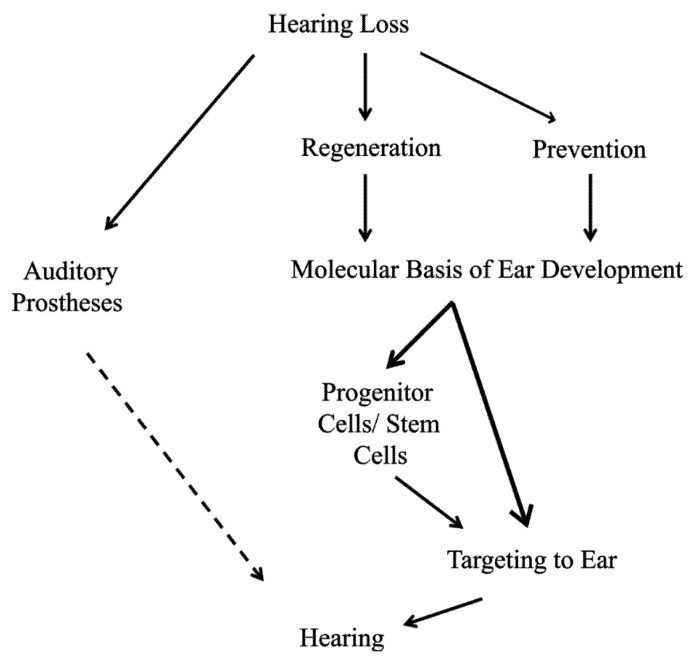
Hearing loss is a major global problem and patient options are limited. Current treatment to restore hearing is centered on the use of auditory prostheses; however, no current option is able to fully restore normal hearing (dashed line). Thus, perfect solutions to this growing problem include prevention of hair cell loss and when hair cell loss does occur, regeneration of damaged tissue is needed to restore full functionality. To successfully prevent the loss of hair cells or regenerate hair cells after loss, we must have a greater understanding of the molecular basis of ear development, specifically, what genes are needed to form and to maintain hair cells. Upon elucidating these minimal essential factors, it may be possible to directly target inner ear tissues. In the case of prevention, increasing the hair cell’s natural maintenance mechanisms may be sufficient. Once hair cells are lost, manipulation of inner ear supporting cells may be possible to form new hair cells. On the other hand, an alternative source of cells, such as stem cells could be first manipulated to form hair cell-like cells and then targeted to the damaged tissue to restore hearing.

Aside from the use of supporting cells, pluri- or multipotent stem cells could be used as a source of precursor populations [33,34]. Transforming a stem cell into a hair cell-like cell requires recapitulating the normal developmental process from naïve ectoderm to neurosensory precursor to differentiated hair cell [47], with the first several steps performed *in vitro* prior to seeding the damaged cochlea. Contextual clues remaining in the cochlea may be sufficient for final placement and differentiation of the neurosensory precursors; however, only a few markers that originally define the organ of Corti and differentiate hair cells remain later in life [27,48] and may not prove sufficient for hair cell placement and differentiation. Indeed, recent data on the use of *Atoh1* misexpression support the conclusion already reached in earlier work, namely that hair cell formation can be initiated but the hair cell may not be long-term viable and certainly cannot be induced after a certain stage in postnatal development [49]. In addition, both under- and overexpression of *Atoh1* can result in hair cell death [28,50]. In summary, regeneration avenues using forced cell cycle re-entry or stem cell manipulation are only feasible if proliferation in the inner ear is understood to the level needed to manipulate these two pathways successfully (Figure 2). 

## 3. Proliferation Is Essential to Regeneration of Organ of Corti Hair Cells

Attempts to regenerate lost hair cells recapitulate to a variable degree normal inner ear development and thus necessitate that normal development is understood to the extent that critical steps can be incorporated into attempts to regenerate hair cells. Surprisingly, one of the least studied networks in organ of Corti hair cell formation is the delicate control of proliferation that is essential for transforming a handful of naïve ectodermal cells into countless differentiated cells. Understanding this process and how it fits into overall ear development may be the critical step in both forming precursor populations of the proper size and forcing cell cycle re-entry, in either case, priming cells to regenerate functional hair cells from damaged tissue and restore hearing. This aspect will be expanded upon in the next paragraphs. 

### 3.1. Ear Development Is a Controlled Balance of Proliferation and Differentiation

The development of the inner ear is dependent upon the robust regulation of proliferation and differentiation that transforms a small patch of undifferentiated ectodermal cells adjacent to the hindbrain into a complex, three-dimensional, functional labyrinth that perceives a wide frequency of sound and participates in multisystem integration to coordinate balance. Concomitant and interdependent with proliferation and subsequent differentiation are otic placode induction, organ of Corti specification, and neurosensory cell formation and maintenance.

### 3.2. Otic Placode Induction

Prior to embryonic day 8 (E8) in mouse, the ectodermal cells adjacent to the hindbrain have no known molecular signature destining them to become the inner ear. At this point, *Pax8* and *Eya1* are expressed, but their roles are not fully clarified [51,52,53]. Subsequently, diffusible factors including the Fgfs and Wnts from the hindbrain and surrounding mesenchyme induce a patch of these ectodermal cells to become neuroectoderm, and the presumptive inner ear, known as the otic placode, becomes defined [54]. This induction process, consisting of mitogenic signals, begins a molecular cascade that transforms the flat otic placode into the otic vesicle and converts ectodermal cells into otocyst cells. During the ensuing invagination process, neuroectodermal precursor cells become specified, multiply and the axes of the otic vesicle are defined, with the ventral compartment housing the cochlea, defined by *Shh* signaling, and the dorsal compartment developing into the vestibular system, controlled by the *Wnts*, *Fgfs* and *Bmp4* [55,56]. These early induction steps encode the molecular identity of the inner ear as mutations in many of these genes result in severe developmental disruptions [57].

### 3.3. Organ of Corti Specification

The molecular cascade that begins the induction and invagination processes that result in the formation of the otic vesicle are essential to the identity of the organ of Corti. *Bmp4*, the *Fgfs*, *Shh*, and the *Wnts* specify the boundaries of and make a “topological blueprint” for the forming organ of Corti [58,59]. These genes transform the neuroectodermal cells of the otic vesicle into competent, yet still proliferating, neurosensory precursor cells [55].

### 3.4. Neurosensory Formation

**Figure 3:**
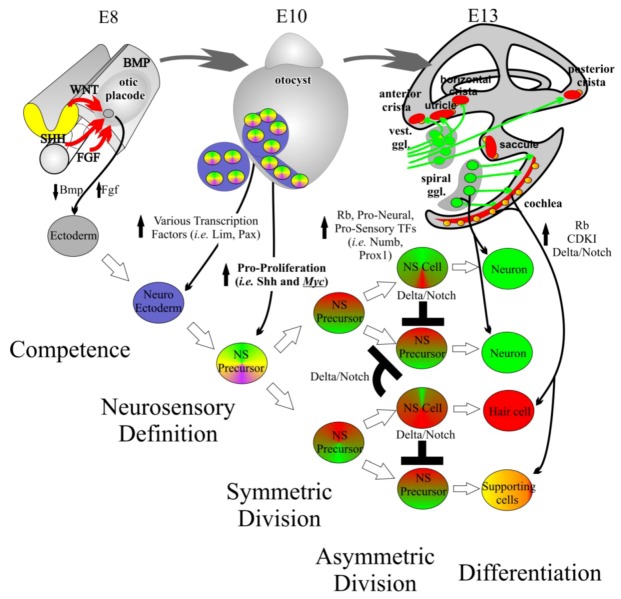
Inner ear development requires controlled proliferation. The inner ear begins as an undifferentiated patch of ectoderm adjacent to the hindbrain at embryonic day (E) 8. Through induction by diffusible factors such as the Wnts and Fgfs, neuroectoderm is formed. From this neuroectoderm, neurosensory (NS) precursor cells are formed. NS precursors have the ability to form neurons, hair cells, and supporting cells. An initially small population of NS precursor cells undergoes symmetric division leading to additional NS precursor cells. This multiplication is in part driven by proto-oncogenes such as the *Mycs*. At a certain point, tumor suppressor genes, pro-sensory genes, and pro-neural genes increase leading to the asymmetric division of NS precursor cells into differentiated NS cells. The formation of NS cells such as neurons and hair cells help maintain the differentiated state of nearby supporting cells. The differentiated state is further maintained by high levels of cyclin dependent kinase inhibitors (CDKIs) and retinoblastoma (Rb). Modified from [33,55,60].

Neurosensory precursor cells retain the ability to form neurons, hair cells, or supporting cells [55] while at the same time these populations increase in size. Controlled proliferation of neurosensory precursors through symmetric and asymmetric division followed by differentiation gives rise to each of these three cell types formed in the correct location, specific orientation, and proper number [60] (Figure 3). Subsets of basic Helix-Loop-Helix (bHLH) transcription factors (TFs) define the formation of normal sensory neurons and hair cells from neurosensory precursors from the same lineage relationship [55].

Many mouse models have attempted to elucidate the molecular basis of ear development and have thus far suggested that (A) there must be a balance of proliferation and cellular differentiation [61], (B) once in a quiescent state, the formation of a sensory neuron and a hair cell from a neurosensory precursor cell is based on a hierarchical decision making process that is dependent upon three bHLH TFs, Neurog1, Neurod1, and Atoh1 [27,28,55,58,62], (C) supporting cells are stabilized by interactions with newly differentiated hair cells through the Delta/Notch pathway to regulate neurogenic genes [58,63,64,65], and (D) hair cells are maintained, in part, by several downstream genes to *Atoh1*, including *Pou4f3* [2,30] and *Barhl1* [31]. Disruptions in many of these steps lead to hair cell loss. Despite tremendous progress in the past two decades, this decision making process is not yet defined to the level that will enable translational medicine to provide therapeutic options to those suffering from hearing loss [66]. As discussed earlier, it is the understanding of the initial balance of proliferation and differentiation (from which the remainder of steps is incumbent upon) that is perhaps the most important in providing long-term therapeutic intervention; yet, it remains sparsely studied. 

## 4. The Complex Balance of Proliferation and Differentiation Is Not Well Understood in the Mammalian Inner Ear

The balance of proliferation and differentiation is life-long and necessary for the proper development and function of specialized cells [55,67,68,69]. Understanding the mechanisms behind this balance is an essential question to developmental biology, cancer biology, and regenerative medicine as this balance defines the development of any organism when adequately regulated but leads to cancer when deregulated and leads to regeneration when properly manipulated. In some tissues, this balance is easily shifted, such that in response to injury, cells are able to re-initiate the cell cycle to produce new cells and functionally replace the damaged tissue. The mammalian inner ear is on the far side of this balance and once cells become post-mitotic they are unable to re-enter the cell cycle naturally, perhaps due to the complexity of the inner ear, which allows for robust hearing that may be disrupted if proliferation were re-initiated.

Therapies towards regeneration of the complex hearing organ demand an understanding of how the balance shifts from a mitotically active to a post-mitotic state with subsequent cellular differentiation during normal development. There may be an intricate interconnectedness in both space and time between key pro-proliferation factors (*N-Myc* and *L-Myc*) and pro-differentiation factors (*Neurod1* and *Atoh1*) in the ear [55], such that disruption in proliferation disrupts proper differentiation. This is supported by various interactome networks elsewhere in the body [70] which have shown that dynamic modular structures regulate the balance of proliferation and differentiation [68].

Proliferation is modulated by redundant cell cycle checkpoints. At each cell cycle checkpoint, there exists a relationship between regulatory machinery responsible for proliferation, including the cyclins, cyclin dependent kinases (Cdks), Cdk inhibitors such as the Kip/Cip and Ink families, tumor suppressors such as the retinoblastoma protein family, and proto-oncogenes. Each of these individual units have multiple and partially redundant subunits (*i.e.*, the cyclins are broken in various classes and subclasses: cyclin A1, cyclin A2, cyclin D1, and cyclin D2). Additionally, each cell cycle checkpoint has a unique function and a unique set of associated factors. To highlight the complexity of proliferation regulation, we look at the G1/S checkpoint which gates the quiescent cell in the G0/G1 phase from undergoing nuclear DNA replication. At this checkpoint, negative and positive signals regulate the phosphorylation state of the Retinoblastoma protein (pRb) and related pRbl1 and pRbl2 [45]. In the unphosphorylated state, pRb binds the E2F family of proteins. When pRb is phosphorylated, the E2Fs are freed to bind to the promoter region of S-phase promoting genes and the cell continues into S-phase. Proteins that promote the phosphorylation of pRb are proto-oncogenes. Here, a number of upstream growth factors modulate levels of cyclinD and cyclinE as well as cdk2, cdk4, and cdk6. Regulation of proto-oncogenes is through tumor suppressors including the Kip/Cip and Ink families. Thus, proto-oncogenes and tumor suppressors are embedded in a complicated network which integrates a number of mitogenic signals forming feedback loops to control the level of mitotic activity in the cell. 

In the ear, the cell cycle dynamics are likely similar as elsewhere in the body but the full mechanisms have yet-to-be worked out. What is known is that the phosphorylation state of cochlear pRbs [45,46,71] is controlled by the pro-proliferative cyclinD family (importantly cyclinD1 and cyclinD2) which are kept in check by the redundant Ink (p15^Ink4b^, p16^Ink4a^, p18^Ink4^, p19^Ink4d^) and Kip/Cip families (p21^Cip1^ and p27^Kip1^), [72,73]. In the ear, the Kip/Cip proteins are expressed beginning at E12.5 in the apical tip of the cochlea [74] immediately after cell cycle exit in the apex and subsequently spread to the base of the cochlea such that cells throughout the cochlea exit the cell cycle from E12.5 in the apex to E14.5 in the base [75]. Manipulating the cyclinD or Kip/Cip families modifies the dynamics of the inner ear cells’ cell cycle to varying degrees but induces DNA damage pathways and cell death [76]. For example, null mice lacking p27^Kip1^ contain cells that are able to stay in the cell cycle longer than control cells, generating supernumerary hair cells; however, these cells are not functional and die [77,78]. Likewise, overexpression of cyclinD1 in supporting cells is able to induce transient cell cycle re-entry [79], but cell cycle re-entry had poor yield.

While direct alteration of the phosphorylation state of pRb is one method to manipulate cell cycle dynamics, manipulations of mitogenic signaling at the cell surface has also been performed [80,81,82,83]. The *Fgfs*, *Tgf*-β, *Shh*, and *Wnts*, many of the genes necessary for the early induction and identity of the inner ear, play later roles in patterning and proliferation of the ear. Manipulations of these ligands and receptors resulted in vestibular cell cycle re-entry in early stages of development but this capacity was lost at later ages [84], suggesting regulation at this level is likely not sufficient for late or long-term cell cycle re-entry. In summary, despite the known importance of proliferation and differentiation, the complex network of the inner ear cell cycle remains mostly enigmatic, yet it represents a source of great therapeutic potential for hair cell regeneration that requires further exploration. 

## 5. The *Mycs* Are an Important Node Integrating Proliferation with Cellular Differentiation

**Figure 4:**
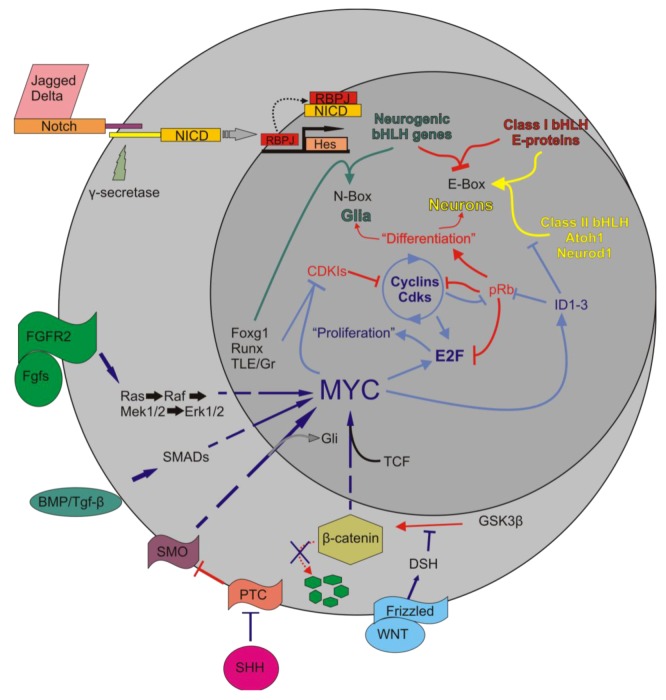
The interactions of *Myc* in the body are complex. Upon activation of Notch by Jagged or Delta, the Notch IntraCellular Domain (NICD) is cleaved by γ-secretase. NICD translocates to the nucleus to form a complex with RBPJ to positively regulate the transcription of the Hes family of bHLH TFs [63]. Myc is regulated by a number of pathways, including the Wnt/β-catenin pathway, Tgf-β or Bmp/Smad, Fgf/Erk1/2, and the Shh/Smo pathways. Once activated, Myc interacts with partner proteins to regulate a number of downstream effectors, including the cyclins, E2Fs, IDs, CDKIs, and various microRNAs. This regulation leads to the inhibition of class II bHLH TFs from binding to the E-proteins which then allows the Hes family to occupy the E-proteins. It also leads to the removal of pRb from E2F and allows E2F to bind S-phase promoting genes. The larger circle represents the cell as a whole while the smaller circle is the nucleus. Arrows indicate upregulation while blunted lines represent inhibition. Red indicates pathways favoring differentiation, blue indicates pathways favoring proliferation. Modified after [33,55,60,89].

Due to the cell death seen with manipulations of downstream genes and lack of late forced cell cycle re-entry with upstream pathways, we aimed to target a node that not only incorporated the upstream pathways and synergized them into the downstream pathway, but one that could also interact with cellular differentiation. As previously stated, bHLH TFs are needed for the differentiation of sensory neurons and organ of Corti hair cells. Some bHLH TFs antagonize cellular proliferation and induce differentiation [85,86,87], indicating that bHLH TFs are able to simultaneously manipulate both pathways. Furthermore, **I**nhibitors of **D**ifferentiation and DNA binding (ID) are known to repress bHLH TFs driving differentiation by binding class II bHLH TFs (*i.e.*, Neurod1 and Atoh1) and inhibiting their DNA binding or directly promoting proliferation by binding pRb to free E2F [88]. Abundant work in other systems identified the bHLH TF Myc as a potential node that synergizes the upstream pathways and indirectly modifies the cell cycle through regulation of the cyclins and Kip/Cip family as well as interacts with the IDs (Figure 4). 

Prior to 2011, our knowledge of *Myc* in the inner ear was limited and the following descriptions of *Myc* interacting partners remain to be confirmed in the ear. It is this very gap in knowledge compared to what is known about the *Mycs* elsewhere in the body to the role it may play in the inner ear that prevents the safe manipulation of *Myc* for therapeutic benefit in patients with hearing loss. To this end, many genes expressed in the ear play a different function elsewhere in the body (for example, *Atoh1* initiates differentiation of hair cells in the ear but controls proliferation in the cerebellum), making a detailed functional study of the *Myc* network necessary before any translational application can be attempted.

### 5.1. Myc Is Tightly Regulated to Ensure a Properly Synchronized State

*Myc* plays a role in virtually every cellular activity [90,91,92] but its main function is in cellular proliferation. Each of the three *Mycs*: *C-Myc*, *L-Myc*, and *N-Myc*, are highly conserved as separate genes across large phylogenetic distances [93,94,95,96]. They have initially overlapping expression during early development with later separation. *C-Myc* has a broad expression profile whereas *N-Myc* and *L‑Myc* are more restricted to specific tissues, most notably the brain, kidney, and inner ear [97]. Despite this, the *Mycs* can often fully functionally substitute when replaced with another *Myc* [96]. The *Mycs* have similar genetic structures, each with three exons with large portions of the first and third exons containing untranslated regions that carry transcriptional or post-transcriptional regulatory sequences [96,98]. 

*Myc* is regulated at every level of processing [99,100,101]. While cis-acting regulatory elements are difficult to elucidate in part due to reliance on non-canonical cis-elements, *Myc* responds to many traditional trans-acting factors such as many of the mitogenic signals present in ear development [92]. *Myc* is silenced through phosphorylation at more than a dozen serine and threonine sites [102,103]; however, the dynamics of *Myc* gene induction and shut-off are incompletely described [92]. 

### 5.2. Myc/Max/Mad Network Balances Proliferation in a Cell

The three Mycs bind to promoter sequences of nearly 1,700 genes (myccancergene.org). Myc does not homodimerize under normal conditions and rarely binds sequence specific DNA in isolation [104]. Myc heterodimerizes with Myc Associated factor X (Max) and binds to DNA with its C-terminal domain and regulates transcription with is N-terminal domain [105]. Myc competes with antagonists Mnt, Mxd, Mga, and the rest of the Mad family, which are highly expressed in differentiated cells, [106,107,108] for binding to Max. Both sets of heterodimers bind the same target genes [109]; however, the accessory binding proteins differ [110]. 

**Figure 5:**
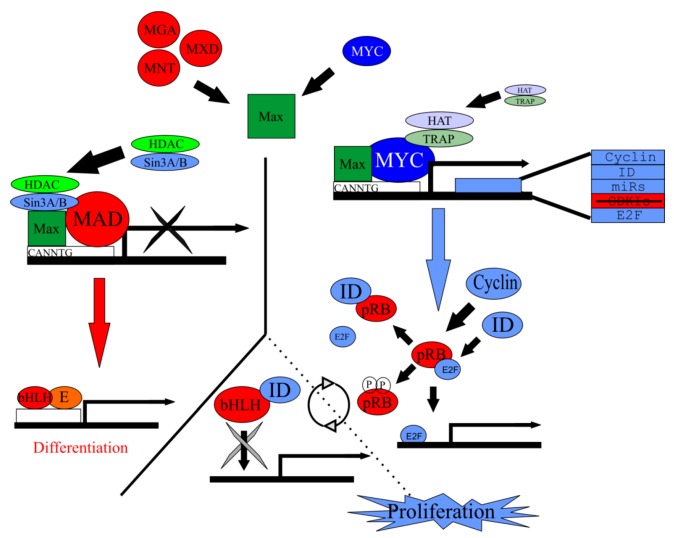
*Myc/Max/Mad* network regulates proliferation. Max forms a heterodimer with Myc but is also capable of forming a heterodimer with the Mad family of antagonists. Either heterodimer will bind to the CACGTG E-box sequence on target genes. Along with accessory binding partners, *Myc/Max* will allow for the transcription of target genes responsible for to proliferation. Contrarily, *Mad/Max* will inhibit the transcription of these target genes, leading ultimately to differentiation. Target genes of *Myc*/*Max* include *cyclin*, *ID*, *E2F*, and various microRNAs. Both the cyclins and IDs remove pRb from E2F and allow E2F to bind to S-phase promoting genes and allow subsequent proliferation. IDs are able to bind bHLH TFs but since they lack a basic motif, are unable to bind E-boxes, inhibiting differentiation. In the absence of IDs or cyclins, bHLH TFs are free to bind to E‑proteins and the E-box, promoting differentiation. Modified from [89].

When the Myc antagonists of the Mad family bind Max, the resulting heterodimer localizes to the *Myc* E-box promoter region (5'-CACGTG-3') of target genes and recruits co-factors Sin3 and components of the histone deacetylase complex (HDAC) to repress the expression of downstream pro‑proliferative E2Fs, cyclinDs, and ID proteins [111,112]. In this repressed state, class II bHLH TFs Neurod1 and Atoh1 are free to heterodimerize to class I bHLH TFs and differentiate post-mitotic neurosensory precursors into neurons and hair cells, respectively, by binding to E-boxes with somewhat similar binding motifs (Figure 5). 

When Myc binds Max, the resulting heterodimer creates an architectural scaffold allowing the binding of co-factor TRAP and components of the histone acetyltransferase complexes (HAT), which together bind with the E-box of the promoter regions of the E2Fs, cyclinDs, IDs, and many other Myc regulated genes to promote transcription [113,114]. Interestingly, Myc/Max has been shown to bind to non-canonical E-boxes containing variant base sequences [115,116], suggesting a potential cross‑talk between bHLH TFs; however, binding affinities to these non-canonical E-boxes vary [117]. Regardless, the *Myc/Max/Mad* network may play a role in both proliferation and differentiation in the ear as they have been shown to elsewhere [117]. Given the complexity and the importance of precise timing during the development of the inner ear, if these relationships hold true, the insights gathered from *Myc/Max/Mad* network manipulation during ear development may prove immensely critical in any future therapeutic intervention, but manipulations of which gene (s) would provide the best insight into this network?

### 5.3. Myc/Max/Mad Is a Highly Conserved Regulator of Proliferation

A glimpse into evolutionary history may provide an ideal candidate gene. The last common ancestor linking invertebrates and vertebrates was about 1 billion years ago [118]. In all organisms, the *Myc/Max/Mad* network is important in proliferation control, but each of the three gene families have a unique evolutionary history that go back to single-celled organisms. All multicellular animal species analyzed contain a single *Max* ortholog which marks the most highly conserved element of the *Myc/Max/Mad* network [119]. Vertebrates contain three *Mycs* that are highly conserved at the important functional motifs [120]. To this end, *dmyc* in *D. melanogaster* and the *Mycs* in vertebrates have conserved functions in both control of growth and proliferation [121] such that *H. sapiens* (human) *Myc* can fully compensate for *dmyc* loss [122]. In support of an early divergence of the *Mycs*, *N-Myc* in *M. musculus* (mouse) has 65% sequence similarity to *N-Myc* in *X. laevis*; however, *N-Myc* in mouse has only a 35% sequence similarity to *C-Myc* in mouse [95,123] indicating that distinct factors evolved differently after their split and that the individual *Mycs* are more highly conserved individually than between members. The *Mad* family is the least well-conserved and consists of five genes in both human and mouse but only one gene in *D. melanogaster* and *C. elegans* [124,125]. Thus, *Mad* gene duplication may have also occurred early in chordate development [119]. With this, it appears that *Max* would be the ideal candidate gene as there exist only one gene that is highly conserved rather than multiple, less well-conserved genes; however, current mouse models do not permit *Max* assessment due to early lethality.

## 6. Ear Specific Knockouts of *N-Myc* and *L-Myc* May Provide Translational Insights

Loss of function studies using mouse knockouts coupled with the recently stated evolutionary history of the *Myc/Max/Mad* network help elucidate the importance of each gene. *Max* knockouts were embryonic lethal by E6.5 [126] and no conditional *Max* mice are currently available but are ultimately needed to eliminate all *Myc* and *Mad* signaling. *N-Myc* null mice were embryonic lethal by E10.5 [97,127,128,129]. This suggested that either *N-Myc* is dispensable until E10.5 or that *C-Myc* function was redundant until this time. Consistent with the latter, prior to E10.5, *N-Myc* and *C-Myc* were co‑expressed but at E10.5, they began to have unique expression patterns [97]. Due to the embryonic lethality of the *N-Myc* null mice, an *N-Myc* floxed mouse was generated [130]. 

*C-Myc* null mice were also embryonic lethal at E10.5 similar to *N-Myc* null mice [131] but conditional mice are available. As previously mentioned, *C-Myc* is not specifically expressed in the brain, kidney, or the inner ear [89,132,133]. In stark contrast to *N-Myc* and *C-Myc* null mice, *L-Myc* null mice were viable without a noticeable phenotype; however, *L-Myc* is always co-expressed with either of the two other *Mycs* [134]. Therefore, this lack of phenotype could be due to co-expression and functional redundancy with either *N-Myc* or C*-Myc*. *L-Myc* conditional knockout mice are also available. Interestingly, of the three *Mycs*, *L-Myc* appears to be the most well-conserved despite its seemingly lesser importance. Knockouts of each of the five individual *Mad* family members resulted in mild phenotypes [97]. In summary, to assess the *Myc/Max/Mad* network regulation in the inner ear, and its role in proliferation and differentiation, conditional knockout lines must use a selective ear specific promoter to drive *Cre* recombination in the currently available *N-Myc* and *L-Myc* floxed mice. 

Prior to Kopecky *et al.*, 2011 and followed up by Dominguez-Frutos *et al.*, 2011, no functional analyses had been performed and no mechanisms had been proposed for *Myc* in the inner ear, making *N-Myc* and *L-Myc* two completely unexplored wildcards in inner ear development [89,133]. For our studies, we used two previously described *Cre* lines, the *Pax2-Cre* transgenic mouse [135], where *Pax2* is one of the earliest markers of the otic placode [136], and the *Atoh1-Cre* transgenic mouse [75], where *Atoh1* is necessary for formation of hair cells of the inner ear [26]. These lines were crossed with *N-Myc* floxed [130] and *L-Myc* floxed (Dr. Eisenman; Jackson Laboratories) mice to assess the function of the two ear expressed *Mycs* during early inner ear development and later in inner ear hair cells. 

Throughout this review we are following a single leading hypothesis: *Mycs*, in particular *N-Myc* an *L-Myc*, are essential players for inner ear neurosensory proliferation and differentiation.

## 7. Summary of Current Literature of *N-Myc* and *L-Myc* Function in the Inner Ear

### 7.1. N-Myc Is Essential for Normal Inner Ear Development

Our working hypothesis was that the *Mycs* are essential players for inner ear neurosensory proliferation and differentiation. Our data clearly support this hypothesis and show that both *N-Myc* and *L-Myc* are present in the inner ear [89,132]; whereas, the third member of the tripartite *Myc* family, *C-Myc* is very limited, if at all present, and likely plays no role [133]. Prior to 2011, there had been no functional studies of any of the *Mycs* during inner ear development, relegating the *Mycs* to an interesting, yet understudied gene potentially important during inner ear development [89]. To elucidate the effect of *N-Myc* on ear development, we generated the first mouse ear specific conditional knockout (CKO) of *N-Myc*, the *Pax2-Cre N-Myc* CKO mouse, which successfully removed *N-Myc* from the entire inner ear shortly after embryonic day 8.5. In the absence of *N-Myc*, the inner ear had a drastic reduction in size and proliferation, a truncated cochlea with a “circularized” apex, a fused cochlea, saccule, and utricle, an absent horizontal canal, and mixed cochlear and vestibular innervations, both centrally as well as peripherally [89,133]. These findings suggested an importance of *N-Myc* in inner ear development and shed light on potential redundant signaling with *L-Myc*, which was highly co-expressed throughout embryonic development of the inner ear and both of which had a late co-expression specific to inner ear hair cells. This further supported our hypothesis that the *Mycs* signaled through similar pathways in the ear as elsewhere in the body as proliferation was dramatically affected.

### 7.2. N-Myc Is Essential to Proper Functionality of Organ of Corti Hair Cells

Despite severe abnormalities in the inner ear and a reduction in size of the cerebellum and the kidney, several *Pax2-Cre N-Myc* CKO mice survived until at least nine months of age [137]. After the initial differentiation of hair cells in the organ of Corti and continued presence of hair cells at both P0 and P7, all cochlear hair cells were progressively lost starting shortly after the onset of hearing (~P18) and was most severe in the base and the apex of the cochlea. This loss resulted in the *Pax2-Cre N-Myc* CKO mice failing to elicit any auditory brainstem response (ABR) at all ages and frequencies tested. Despite the abnormal morphologic development of the entire inner ear and loss of cochlear hair cells, vestibular hair cells and innervations remained until at least nine months of age [137]. Using three measures of gait performance, the *Pax2-Cre N-Myc* CKO mice suffered from severe ataxia that did not improve with time [137] suggesting functional input and partial compensation. The differential loss of organ of Corti hair cells suggested a greater importance of *N-Myc* to the formation and survival of cochlear compared to vestibular hair cells. Furthermore, the *Mycs* must play a major role in hair cell viability, either directly or indirectly through the altered morphology they generate. For example, it is possible that the lack of a ductus reuniens and utriculo-saccular foramen allows a broad open communication between scala media and vestibular endolymph. This could result in altered ionic concentrations that are more detrimental to organ of Corti hair cells, known to respond to ionic changes very sensitive.

## 8. Neither *N-Myc* nor *L-Myc* Are Essential for the Long-Term Survival of Organ of Corti Hair Cells

The observation that organ of Corti hair cells were lost after initial formation indicated that *N-Myc* was critical for either (A) the long-term maintenance of organ of Corti hair cells or (B) the initial formation of these hair cells. In support of A, both *N-Myc* and *L-Myc* were found specifically in hair cells at P0, long after cell cycle exit (E14.5) and differentiation of hair cells was completed. Furthermore, the half-life of *Myc* mRNA is approximately 30 minutes [138,139], indicating that the mRNA expression seen at P0 is a secondary upregulation specific to inner ear hair cells. To address how the loss of *Myc* affected long-term viability of hair cells, we needed to knock out *Myc* early in hair cell precursors and later specifically in hair cells. However, since knocking out the *Mycs* early in development would necessarily knock them out later in hair cells, we needed to first knock out the *Mycs* specifically in hair cells to help segregate the early and late function of the *Mycs* on hair cells. To critically test the importance of *N-Myc* and *L-Myc* for the long-term maintenance of hair cells, we generated an *Atoh1-Cre N-Myc f/f L-Myc f/f* mouse that knocked out *N-Myc* and *L-Myc* in all hair cells after hair cell differentiation, which is dependent upon *Atoh1* [26,140]. In all behavioral, histological, and morphological analyses, the *Atoh1-Cre N-Myc f/f L-Myc f/f* mice had no measurable abnormalities, suggesting that neither *N-Myc* nor *L-Myc* are important for long-term survival of hair cells and that the late-onset loss of hair cells is likely due to an initial insult resulting from the early loss of the proto-oncogenes disrupting the timing of proliferation and differentiation [137]; however, this hypothesis needs to be further assessed. This is the first indication that late-onset loss in humans could potentially be a result of a much earlier-onset mutation predisposing to the loss of hearing and furthermore, indicating that proper timing during the transition from proliferation to differentiation may result in late-onset deficits. Other known genes showing a delayed loss of hair cells such as *Pou4f3* [2,30] or *Barhl1* [31] have only expression in maturing hair cells and thus do not show a biphasic expression as the *Mycs* and are more easily reconcilable with the delayed-onset loss of hair cells. Future work is needed to define the molecular alterations in the normally developing hair cells of the *Pax2-Cre N‑Myc* CKO mice that result in their belated demise.

### N-Myc and L-Myc Are Necessary for Proper Neurosensory Developmental Timing

Given the absence of an effect when *N-Myc* and *L-Myc* are removed in differentiated hair cells, we aimed to further determine the effect on inner ear development in the early absence of *N-Myc*, *L-Myc*, or both. To assess this early effect of *N-Myc* and *L-Myc* on neurosensory development, we knocked out both *N-Myc* and *L-Myc* during early development in the entire ear using *Pax2-Cre* [141]. Resulting mice had a more severe phenotype than the *Pax2-Cre N-Myc* CKO mice suggesting that *N‑Myc* and *L‑Myc* were partially redundant. However, when *L-Myc* was knocked out, *N-Myc* was able to nearly fully compensate for this loss. On the other hand, when *N-Myc* was knocked out, *L-Myc* was unable to compensate, illustrating a greater importance of *N-Myc* than *L-Myc* in inner ear development, consistent with our initial hypothesis. To analyze the combined effect on the timing of proliferation and differentiation in the absence of both *N-Myc* and *L-Myc*, we assessed proliferation using both EdU, a thymidine analog that incorporates into DNA during replication, and qRT-PCR. We also used qRT-PCR to assess relative levels of genes that are important in differentiation [141].

Between E10.5 and P0, *Pax2-Cre N-Myc f/f L-Myc f/f* mice had a reduction in proliferation and a decrease in cellular differentiation bHLH mRNA levels of *Neurod1* and *Atoh1* along with *Atoh1* downstream and cell maintenance genes *Pou4f3* [2,30] and *Barhl1* [31]. This alteration in timing of cell cycle exit and cellular differentiation along with decreased levels of cell maintenance genes may be to blame for many of the malformations seen in the inner ear, including the progressive loss of organ of Corti hair cells. However, further analyses of gene binding and localization of gene expression patterning changes should be done to confirm these findings.

## 9. Conclusions

**Figure 6:**
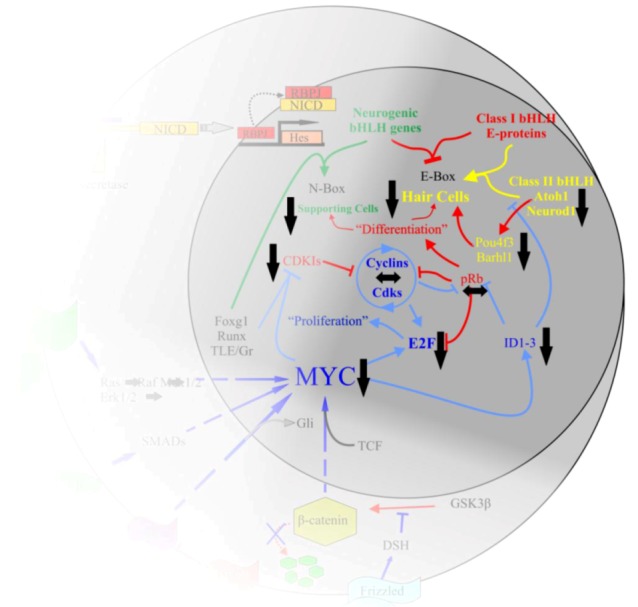
The *Myc* interactions in the inner ear require additional clarification. We began our exploration of *N-Myc* and *L-Myc* function following our stated hypothesis that *N-Myc* and *L-Myc* were important to proliferation and possibly followed similar pathways in the inner ear as the *Mycs* had been described to elsewhere in the body. While the full mechanisms require further elucidation, we have provided some clarity to the picture. *N‑Myc*, and to a lesser extent, *L-Myc* are important to inner ear development. In the absence of *Myc*, *E2F*, *ID*, *CDKI*, *Atoh1*, *ND1*, *Pou4f3*, and *Barhl1* are all reduced. cyclinD2 and pRb appear not to be greatly affected. This leads to a progressive loss of the organ of Corti hair cells. Figure modified from [55,60,89].

The early embryonic losses of *N-Myc* and *L-Myc* in the inner ear resulted in numerous morphologic and histologic abnormalities. These developmental malformations resulted in functional deficits and late-onset hair cell loss. In the ear, *N-Myc* appeared to be the most important *Myc* and likely regulates many interconnected pathways; however, the *Mycs*’ roles appeared to be essential only during early development as *Myc* loss late during development had little to no effect on hair cell function or organ of Corti development. Importantly, the early loss of *N-Myc* and *L-Myc* resulted in decreased proliferation and an alteration in the relative levels of important genes essential for cellular differentiation. Despite these novel findings, there remain many unanswered questions (Figure 6). In the next section, we will discuss what these findings could potentially mean as we strive for finding a permanent solution to hair cell loss and what additional work must be performed to obtain the ultimate cure to hearing loss.

## 10. Translational Significance and Future Directions

### 10.1. Hearing Loss Prevention Is Key, but N-Myc and L-Myc Are Unlikely to Play a Role

As mentioned in the introduction, the simplest and most effective way to solve hearing loss is to prevent the onset of hair cell loss. In cases where the hair cell loss is genetic, the genetic defect must be corrected to prevent the loss of hearing; however, in many other cases where the loss of hair cells is due to an accumulation of environmental insults, enhancement of a hair cell’s maintenance pathways may be sufficient [142]. By knowing the genes necessary for hair cell formation and maintenance, it may be possible to artificially increase endogenous levels of a subset of these genes, whether that is through gene therapy or pharmacologic intervention (*i.e.*, taking a pill prior to anticipated exposure to insult), to prevent the loss of hearing. Prevention of hair cell loss is especially important given the enormous complexity of regenerating the very delicate organ of Corti [34,58]. We have described the *Pax2-Cre N-Myc* CKO mice, which showed late-loss of cochlear hair cells, coupled with the unexpected secondary upregulation of *N-Myc* and *L-Myc* in differentiated hair cells [89,137]. These findings shed light on a possible auxiliary role of the *Mycs* in hair cell maintenance. This coupled with *Myc’s* known functionality in numerous pathways outside of proliferation such as metabolism, cell death, and biosynthesis, made *N-Myc* and *L-Myc* intriguing candidates for long-term hair cell maintenance studies. 

Unfortunately, follow-up studies showed that in the absence of *N-Myc* and *L-Myc* in hair cells after hair cell differentiation, no differential phenotype was noted, both over time and in the presence of the known ototoxic drug cisplatin [140]. Prior to ruling out *N-Myc* and *L-Myc* in the therapeutic prevention of hair cell loss, *N-Myc* and *L-Myc* overexpression experiments should be performed. Overexpressing these two *Mycs* in hair cells and then subjecting them to noise or chemical induced deafening protocols followed by behavioral tests and histological analyses are needed to conclusively state *Myc’s* role in preventative medicine. Overexpression of both *Mycs* could be performed through either a genetic mouse model, such as the existing Smo overexpressor crossed with *Atoh1-Cre* or a tamoxifen-inducible *Cre*, or with viral transfection of *Myc*, similar to what is currently performed for induction of pluripotent stem cells [143]. Regardless of the role the *Mycs* play in hair cell maintenance, they are likely to be important in regenerative pathways including forced cell cycle re-entry of endogenous cells and in any avenue attempting to use exogenous stem cells.

### 10.2. Proper Manipulation of N-Myc and L-Myc Is Likely Required for Regenerating Hair Cells

Regeneration of lost hair cells may be accomplished through endogenous (*in vivo*) or exogenous (*in vitro*) therapies. *In vivo* approaches force inner ear supporting cells to re-enter the cell cycle followed by directed terminal differentiation into hair cells and supporting cells using viral vectors, including adeno-associated virus serotype 2 or adenovirus serotype 5 [33,144,145,146,147,148]. *In vitro* approaches use stem cell populations and culture them into hair cell-like cells and seed hair cell-like cells onto the cochlea [47,149]. The pros and cons and technical difficulties of these two approaches are considered in detail elsewhere [33,34]; thus, we will focus on the role the *Mycs* may play in these two translational applications. 

### 10.3. Regeneration of Hair Cells through Forced Cell Cycle Re-Entry of Supporting Cells

Non-mammalian species have automatic repair mechanisms in response to injury to the hair cells of various sensory organs. Upon damage to hair cells, supporting cells re-enter the cell cycle to form a new hair cell and supporting cell. This does not happen in mammals, likely due to the enhanced complexity of the inner ear and the increased variety of hair cells and supporting cells [150]. It is because of this phenomenon that makes hearing loss in mammals irreversible. Many efforts have attempted to “jumpstart” the cell cycle of supporting cells; however, no attempts have resulted in long‑term success. Forced cell cycle re-entry has been shown possible through removal of certain cell cycle inhibitors including pRb [46,151,152] and many of the cyclin dependent kinase inhibitors of both the Ink and Kip/Cip families [44]. Unfortunately, all cells forced to re-enter the cell cycle ultimately die. Cell death may result from an accumulation of DNA replication errors and DNA double-strand breaks as inhibition of p53 tends to ameliorate this damage [44,144], but an exact mechanism is unknown. P53 is thought to be the primary mediator of death of tumor cells and outer hair cells treated with cisplatin [144,153,154]. Interestingly, *Myc* tumor cells inhibit p53 [155,156,157]. It is not known if forced cell cycle re-entry through *Myc* will have the added benefits of reduced p53.

While the roles that *N-Myc* or *L-Myc* may play in forced cell cycle re-entry and amelioration of subsequent cell death is speculative, the *Mycs* are interesting candidates for several reasons:

(1) The *Mycs* are essential, non-redundant, untested proto-oncogenes necessary for proliferation. The inner ear appears to be resistant to cancer formation [144] and therefore provides an ample ground to test post-mitotic cell cycle re-entry either through inhibition of tumor suppressor proteins (*i.e.*, pRb, Ink, Kip/Cip) or an increase in proto-oncogenes proteins (*i.e.*, E7). Despite this, forced cell cycle re‑entry has focused on the inhibition of tumor suppressors with very few studies manipulating proto‑oncogenes and none assessing the *Myc* node, despite its known importance elsewhere in the body. Furthermore, *N-Myc* and *L-Myc* are expressed strongly in the inner ear until at least P0.
(2) *Myc* manipulation may ameliorate cell death mechanisms. Pathways currently being manipulated are essential to the direct progression of the cell cycle and forced cell cycle re-entry is being performed through bypassing normal cell cycle checkpoints, such that damaged DNA that would normally trigger cell cycle discontinuation (through p53), now is propagated until cell death. Cells that re-enter the cell cycle in the absence of p53 have a longer survival [43,44,158]. However, maintaining cell cycle checkpoints (through not removing the Kips/Cips or pRb) may be necessary to ensure the accuracy of newly synthesized DNA. In addition to indirect cell cycle control, the *Mycs* are essential for cellular metabolism and DNA synthesis. *Myc* upregulates both ribonucleotide reductase, which is needed for the synthesis of deoxyribonucleotide triphosphates [159] and serine hydroxymethyltransferase, which is required in nucleotide metabolism [160]. Together, manipulation of the *Mycs* would allow some functioning of the cell cycle checkpoints and the resulting DNA replication may be more robust.
(3) In non-mammalian systems, shortly after hair cell damage, both *Atoh1* and *Notch1* pathways are activated [161,162] indicating a necessity for properly timed onset of terminal differentiation. We have shown that the timing of proliferation and differentiation is in part driven by the *Myc* node and that this timing is necessary for proper neurosensory differentiation as viability of hair cells may depend on a proper delay between cell cycle exit and onset of *Atoh1*-mediated differentiation that varies systematically along the organ of Corti [75]. Forcing proliferation and then inducing differentiation without ensuring that the newly formed progenitor cell populations have had enough time after exiting the cell cycle and are receptive to differentiation is a possible cause of the initial formation of hair cell-like cells followed by hair cell death seen in forced cell cycle re-entry and similar to the pattern that was seen in the *Pax2-Cre N-Myc* CKO. Thus, manipulation of *Myc* to force a post-mitotic cell into cell cycle entry, if possible, may make the transition back into the post-mitotic and subsequent differentiated state more long-lasting, achieving in effect the opposite of what was found in the *N-Myc* CKOs.

In summary, upregulation of *Myc* in post-mitotic cells must be attempted to see if forced cell cycle re-entry can be successful either with upregulation of *N-Myc* and/or *L-Myc* alone, or in combination with inhibition of tumor suppressors. Follow-up experiments should be performed to ensure the proper timing between subsequent cell cycle exit and *Atoh1* (or other genes) upregulation that may be crucial for long-term viability of hair cells.

### 10.4. Regeneration of Hair Cells from Stem Cells

Upon upregulation of *Myc* to force cell cycle re-entry of supporting cells, the cells may have properties similar to, or otherwise act like, a stem cell. Stem cells can also be acquired through induction of skin fibroblasts (pluripotent) [143] or epidermal neural crest stem cells from the bulge region of skin hair follicles (multipotent) [33,163,164] and are characterized by their ability to self‑renew. *Myc* (either *C-Myc* or *N-Myc*) is one of four transcription factors [143] that are able to reprogram fibroblasts into pluripotent stem cells. The *Mycs* play an integral role in maintaining the “stemness” of stem cells [33] such that without the *Mycs*, stem cells become quiescent and begin the transition from a stem cell to a differentiated cell. 

In applying stem cells as therapy, *in vitro* manipulation must mimic the natural *in vivo* scenario as closely as possible prior to inclusion of the stem cell into the damaged ear [47]. This manipulation recapitulates development and must therefore regulate both *N-Myc* and *L-Myc*. The loss of *N-Myc* and *L-Myc* in the *Pax2-Cre N-Myc f/f L-Myc f/f* mice resulted in a reduction in proliferation and an alteration in the timing of differentiation. In stem cell manipulations, the same loss may prematurely truncate cell number and would prohibit proper differentiation. On the opposite end of the spectrum, if the cell cycle is not adequately controlled, tumorigenesis is possible. In summary, the efficient and safe use of stem cells for hair cell restoration depends greatly on the tight regulation of the *Mycs*. To test the feasibility of transforming stem cells into stable hair cells and assessing the role of *Myc* in this transformation, microRNAs regulating *Myc* in stem cells can be used to repress the function of *Myc* and force the stem cells into a quiescent state, which may allow better temporal control of differentiation cues, such as *Atoh1*. 

### 10.5. Further Assessment of Functional Redundancy in the Myc/Max/Mad Network

We have concluded that *N-Myc* can nearly fully compensate for the loss of *L-Myc* but *L-Myc* can only minimally compensate for the loss of *N-Myc*. These findings are consistent with the hypothesized lesser importance of *L-Myc* relative to *N-Myc* or *C-Myc* elsewhere in the body [134]. To our knowledge, this is the first direct assessment of *L-Myc*’s ability to compensate for the loss of another *Myc* as no other models have knocked out *L-Myc*, *N-Myc*, or both in the same litter to assess relative compensation between the *Mycs*. However, to conclusively test our findings, replacing *L-Myc* coding sequences for *N-Myc* coding sequences (and *vice versa*) in the ear, similar to the test that replaced *N‑Myc* coding sequence with *C-Myc* [96], or one proneural bHLH gene by another [62], should be performed. This would show not only the functional role of *L-Myc* in the *Myc/Max/Mad* network, it could also support the hypothesis that *L-Myc* is slowly evolving into a functionally inactive form [97,165,166] and may turn into a “pseudogene” in the future.

There are five *Mad* genes and conditional deletions of all five of the genes and deletion combinations of the five genes may provide additional insight to proliferation regulation in the ear. In 2006, a triple null *Mad1*, *Mxi1*, and *Mad3* mouse had been generated resulting in a viable mouse with slightly increased size [97]; however, no data on the ear phenotype is currently available. While no current *Max* conditional knockout is available, generating a *Max* floxed mouse would render all *Myc* and *Mad* signaling absent. This later experiment may be especially important given the continued expression of *Max* in both proliferating and quiescent cells [167] which may provide a gene, in addition to the *Mycs*, that can be targeted later in life.

Together, the field of hearing research is facing a challenging, yet exciting future. With hearing loss so prevalent and the cost to each patient so great, the challenges we face must be met with renewed enthusiasm, creativity, and vision to ensure that allocated resources have the greatest potential to expedite the transition from bench to bedside. The wave of inner ear regenerative medicine will likely need to consider the roles that *N-Myc* and *L-Myc* play in controlling the balance of proliferation and differentiation. It is this balance that has long been critical for the maintenance of life and there is little doubt that hidden within this balance are the answers that over 500 million people are waiting to hear.
